# Comparing Resection and Primary Anastomosis versus Hartmann's Stoma on the Mortality and Morbidity of Gangrenous Sigmoid Volvulus: Systematic Review and Meta-Analysis

**DOI:** 10.4314/ejhs.v33i6.19

**Published:** 2023-11

**Authors:** Atalel Fentahun Awedew, Zelalem Asefa, Biruktawit Destaw Enkoye

**Affiliations:** 1 Department of surgery, Addis Ababa University, Ethiopia; 2 Redate Health Care, Addis Ababa, Ethiopia

**Keywords:** sigmoid volvulus, gangrenous bowel, Primary resection and anastomosis, Stoma, Hartmann's procedure

## Abstract

**Background:**

Gangrenous sigmoid volvulus has a significant impact on morbidity and mortality. This study was conducted to compare sigmoid resection and primary anastomosis (RPA) with sigmoid resection and end colostomy (Hartmann's procedure) for gangrenous sigmoid volvulus.

**Methods:**

A systematic review and meta-analysis study design was employed to summarize retrospective cohort, prospective cohort, and randomised control trial studies published from inception to march 31, 2023. Searching was performed on Medline, CINAHAL, Web of Science, Google Scholar, the Cochrane Library, and ClinicalTrials.gov to locate eligible articles. Data searching, selection and screening, quality assessment of the included articles, and data extraction were done by two separate reviewers. RevMan 5.4 software with a fixed-effect Mantel-Haenszel model and Stata version 14 were used to analyze the data. The protocol registered on PROSPERO registration website (CRD42023413367).

**Results:**

Ten cohort studies and one randomised control trial with 724 patients were found; all of them were rated as being of moderate quality. The overall mortality after RPA was 15% (95%CI: 11-19%), and after Hartmann's procedure it was 19% (95%CI: 15-23%). Resection and primary anastomosis (RPA) for gangrenous sigmoid volvulus had slightly lower mortality rate than stoma (OR=0.98(95%CI: 0.68-1.42), p=0.07, I^2^=43%), which had no statistically significant difference. Resection and primary anastomosis (RPA) had a slightly higher morbidity rate than Hartmann's procedure (OR=1.01(95%CI: 0.66-1.55), p=0.30, I^2^=18%), which had no statistically significant difference.

**Conclusion:**

Sigmoid resection and primary anastomosis (RPA) and Hartmann's procedure had no significant differences in mortality and morbidity for the treatment of gangrenous sigmoid volvulus. Choice of the intervention for gangrenous sigmoid volvulus should be individualized with consideration of different detrimental factors.

## Introduction

Sigmoid volvulus has a significant impact on morbidity, mortality, health-care costs, and emergency surgical burden. It is the third leading cause of large bowel obstruction in the western world, after colorectal cancer and diverticular disease, but it accounts for 50-80% of large bowel obstruction in the volvulus belt area such as Middle East, South America, Africa and Russia (1-5).

Meso-sigmoid twisting of upto180^0^ in sigmoid volvulus is considered physiological (6). Torsion beyond 180^0^ can lead to colonic ischemia, obstruction and perforation, and only 2% of cases can spontaneously derotate while 70% of cases occur in the counter clockwise direction (7,8).

More than one-third of sigmoid volvulus patients had gangrenous bowel (9). The significant aspect of gangrenous bowel is its high mortality, morbidity, and length of hospital stay (4). Evidence from hospital-based retrospective studies indicated that the incidence of mortality in gangrenous sigmoid volvulus ranged from 17-100% compared to 10-30% in non-gangrenous sigmoid volvulus (10).

Management of sigmoid volvulus depends on the viability of the bowel. For sigmoid volvulus patients with viable bowel and no signs of ischemia or perforations, sigmoidoscopic guided or rectal tube deflation have been advised as the first line treatment (8). Different investigations noted that urgent surgical intervention for sigmoid volvulus is required in 5-22% of patients when the deflation is not possible and 5-25% of patients presented with gangrenous bowel (11).

Urgent sigmoid resection has been advised in cases of gangrenous colon or when sigmoid volvulus deflation is unsuccessful (8). However, bowel continuity following resection has been a topic of debate for surgeons due to lack of highlevel evidences. After removing the gangrenous portion of the sigmoid colon, the decision to perform primary anastomosis or Hartmann's stoma should be individualized, taking into account both the patient's overall condition and the colon (11). As a result, this meta-analysis was conducted to compare resection and primary anastomosis versus Hartmann's stoma on mortality and morbidity among patients with gangrenous sigmoid volvulus.

**Research question**: Is resection and primary anastomosis more effective than stoma at preventing morbidity and mortality when treating gangrenous sigmoid volvulus?

Our meta-analysis question was based on PICOS approach.

Population = adult patient with diagnosis of gangrenous sigmoid volvulus

Intervention = Resection and primary anastomosis

Comparison = resection and primary anastomosis Vs stoma

Outcome = Mortality and morbidity

Studies = Cohort and randomized control trials

## Methods

**Figure 1:**
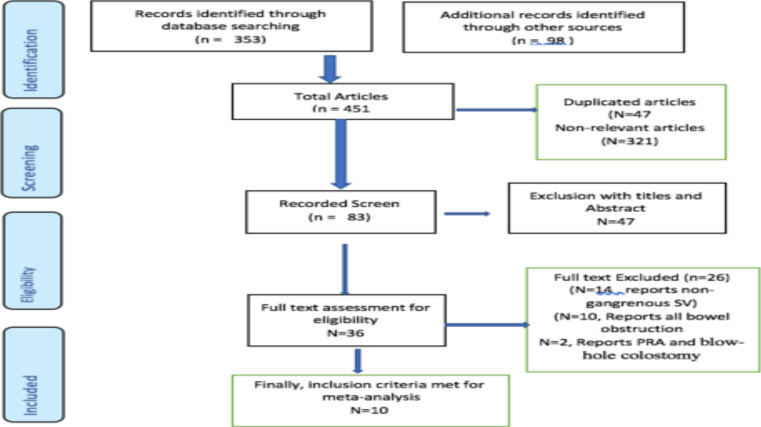
Flow chart for selection of papers. Charts the selection of articles in the review in line with the Preferred Reporting Items for Systematic Review and Meta-analysis (PRISMA) Framework

**Study design**: A systematic review and meta-analysis was conducted to summarize prospective cohort, retrospective cohort, and randomised control trial studies published from inception to march 31, 2023. This review was carried out in accordance with Preferred Reporting Items for Systematic Reviews and Meta-analyses (PRISMA) guidelines (12,13) Figure 1). The protocol of this meta-analysis was registered at PROSPERO **(CRD42023413367).**

**Eligibility criteria**: Retrospective cohort, prospective cohort, and randomized controlled trial studies published from inception to March 31, 2023 on patients with gangrenous sigmoid volvulus who underwent primary resection and anastomosis versus stoma were considered to be eligible for inclusion. Systematic review studies, case reports, case series, and studies with unclearly reported results were excluded from the analysis. The English language restriction and time frame were chosen for convenience and sufficiency for demonstrating a trend of events (14,15)

**Searching strategies and sources**: Searching was performed at Medline, CINAHAL, Web of Science, Google Scholar, the Cochrane Library, and ClinicalTrials.gov to locate eligible articles. Two separate reviewers conducted the electronic data base search and selection of eligible articles. Disagreements between two reviewers were resolved through discussion, consensus, and, if necessary, the involvement of a third party. Our searching strategy was based on the Population, Interventions, Comparison, Outcomes, and Studies (PICOS) approach. Eligible articles were identified using key words and MeSH words in an electronic database, and hand searching using bibliographic or reference information from identified studies. Boolean operator, Wild cards, and splinting words and phrases were employed to widen our search. Terms such as “sigmoid”, “volvulus”, “gangrenous”, “intestinal obstruction”, “bowel obstruction”, “sigmoid volvulus”, “cohort”, “randomized control trial” “stoma”, “Hartman procedure”, and “colostomy” were used to search the eligible articles.

**Screening and study selection**: Two authors independently screened articles using the inclusion criteria. Duplicate studies from various electronic databases obtained through the search strategy were removed using the EndNote program. The titles and abstracts of the articles found through the search strategy were independently screened by two reviewers to eliminate obvious non-relevant papers. The full-text versions of the remaining potentially eligible articles were then retrieved and independently assessed by two reviewers to determine if they met the inclusion criteria. Disagreements are resolved through consensus and discussion. The corresponding author was contacted in order to obtain copies of papers whose full text was not available online. The reasons for exclusion at the full-text screen level were documented in accordance with the PRISMA framework. The total number of unique studies from all sources that meet the inclusion criteria have been recorded.

**Data extraction and management**: Data were extracted using a piloted standardized data extraction form adapted and customized from the Cochrane data extraction of randomized and non-randomized studies (16,17). The publication details, language of the paper, study period, study location, geographic setting, study design, study period, characteristics of participants, sample size and sampling technique, explanatory and outcome variables, data analysis, and the major findings were extracted.

**Risk of bias assessment**: The included studies' qualities were assessed using Cochrane RCT assessing tools that were specifically designed and validated for RCT studies (18). The JBI quality checklist was used for assessing the quality of observation studies (19). As with the study selection, two different investigators independently evaluated the quality of the included papers and characterized them as having high, moderate or low risk of bias.

**Outcome of interest**: Mortality and morbidity were the main outcomes of this review. Mortality was defined as a death that occurred prior to hospital discharge, and morbidity was defined as complication of Clavien Dindo Grades II–IV, which occurred after surgery and before discharge of the patient from the hospital.

**Data synthesis and analysis**: A standard pairwise meta-analysis was preformed using RevMan 5.4 software and STATA version 14. Pooled odds ratios (ORs) with 95% confidence intervals (CIs) were calculated using fixed -effect Mantel-Haenszel model because our results would not make generalization beyond the included studies. Heterogeneity across the included studies were measured using Higgins (I^2^) test, which represents the percentage of variability in effect estimates among the included studies. Higgins (I^2^) test values of 25%-50%, 50-75% and above 75% were considered to have low, moderate, or high heterogeneity, respectively (20,21). Once heterogeneity was identified, sensitivity analysis was carried out by excluding one study at a time and assessing the impact on the final result. Publication biases for the mortality and morbidity was assessed using funnel plot.

## Results

Ten retrospective and prospective cohort studies and one RCT studies involving 724 patients were found, all of which were labeled as having a moderate level of quality (22-31) (Figure 1). All studies reported that gangrenous SV was found predominantly in men. Management of gangrenous sigmoid volvulus is one of the clinical dilemmas due to absent high-level evidences.

Emergency surgical treatment for gangrenous sigmoid volvulus is associated with significant mortality and morbidity, regardless of whether it involves stoma or primary resection and anastomosis (8). Numerous small-scale clinical studies indicate that factors such as age, the presence of shock, comorbid conditions, Ileo sigmoid knotting, delayed presentation, and history of volvulus attacks affect mortality after emergency surgical intervention (8,22,32,33).

**Figure 2:**
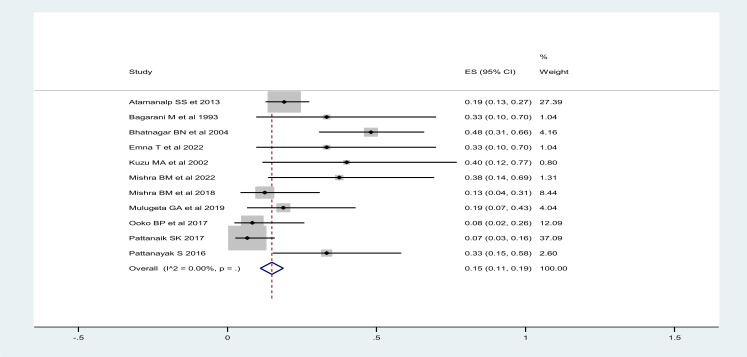
Overall mortality after resection and primary anastomosis for gangrenous sigmoid volvulus

**Figure 3:**
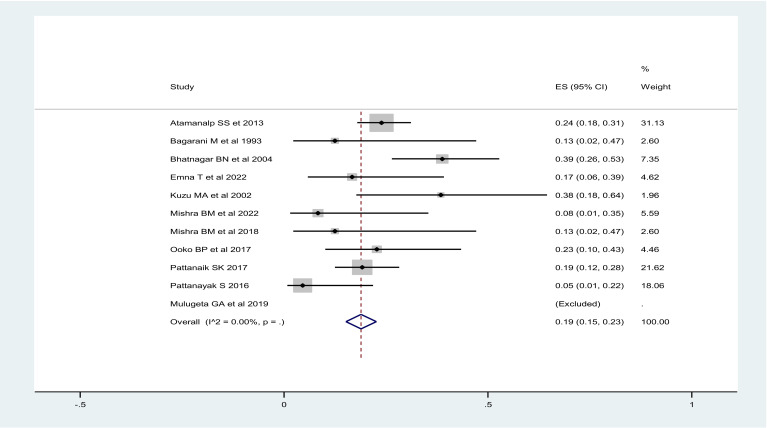
Overall mortality after stoma for gangrenous sigmoid volvulus

The mortality rate for gangrenous sigmoid volvulus was ranging from 9-42%, with overall mortality rate after surgical intervention being 19% (95%CI: 16-22%), regardless of the type of surgical interventions. Mortality after resection and primary anastomosis ranged from 8-48%, with overall mortality after RPA of 15% (95%CI: 11-19%) (Figure 2) and mortality after stoma ranged from 0-39%, with overall mortality rate of 19% (95%CI: 15-23%) (Figure 3).

**Figure 4:**
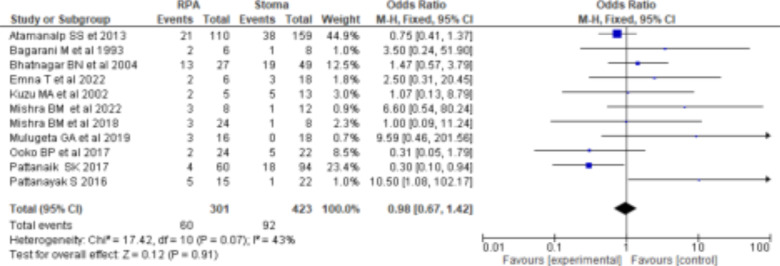
Forest plot comparing RPA and Stoma mortality rates

**Figure 5:**
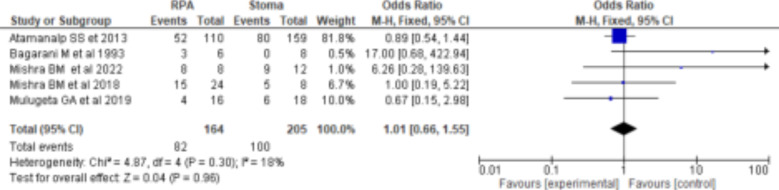
Forest plot comparing RPA and Stoma morbidity rates

**Figure 6:**
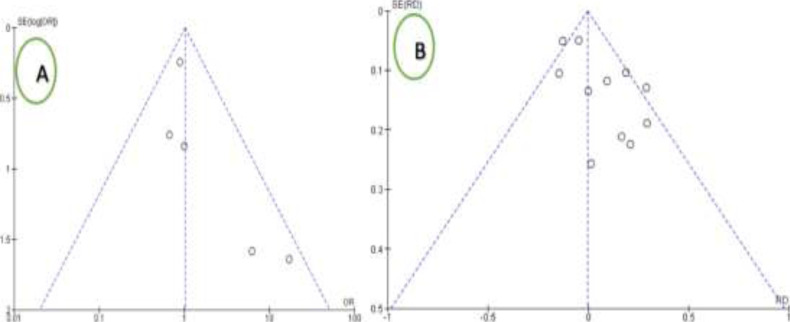
Funnel plot for Morbidity (A) and mortality (B)

The mortality rate for gangrenous sigmoid volvulus ranged from 9-42%, with overall mortality rate after surgical intervention being 19% (95%CI: 16-22%). Resection and primary anastomosis for gangrenous sigmoid volvulus had a higher mortality rate than stoma (OR=0.98(95%CI: 0.68-1.42), p=0.07, I^2^=43%), which had no statistically significant difference (Figure 4). Five studies reported morbidity. Resection and primary anastomosis had a higher morbidity rate than stoma (OR=1.01(95%CI: 0.66-1.55), p=0.30 I^2^=18%), which had no statistically significant difference (Figure 5). Low levels of heterogeneity were found in the included studies (I^2^ for mortality and morbidity was 43% and 18%, respectively) (Figure 4 and 5) with no significant publication biases (Figures 6). ***Figure 4:*** *Forest plot comparing RPA and Stoma mortality rates*.

The included articles did not find any evidence of mortality and morbidity with statistical significance associations with the type of procedure. However, the advice given in each of the articles included was not consistent. Some of the included articles suggested primary resection and anastomosis for gangrenous sigmoid volvulus with specific prerequisites like intraoperative hemodynamic stability, absence of gross contamination, capability to achieve a tensionfree anastomosis, presence of viable and well-vascularized bowel ends after resection, and good functional status (22,24-26,31). In contrast, some of the included studies in this review recommended stoma due to low mortality rates, fear of anastomotic leaks, quick procedure time, and short hospital stays (23,27,30).

## Discussion

To the best of our knowledge, this is the first meta-analysis to provide evidence on the comparative safety of resection and primary anastomosis and Hartmann's stoma for the treatment of gangrenous sigmoid volvulus. This meta-analysis, which included 724 patients, was conducted using ten prospective and retrospective cohort studies and one RCT, which all were of moderate quality. Low levels of heterogeneity were found in the included studies (I^2^ for mortality and morbidity was 43% and 18%, respectively).

The findings of this review revealed that mortality rate after resection and primary anastomosis for gangrenous sigmoid volvulus was approximately 19%, which was fairly close to previous research findings based on clinical data collected over four decades with 393 emergency sigmoid volvulus surgeries, where mortality rate after primary anastomosis was 22% (4).

This study also found that mortality from resection and primary anastomosis and Hartmann's stoma had no statistically significant difference. Gangrenous sigmoid volvulus has a poor prognosis. Evidence from various studies indicated that the mortality rate for gangrenous sigmoid volvulus ranged from 11 to 80% compared to 6 to 24% for non-gangrenous sigmoid volvulus (4,10,33).

We also found that resection and primary anastomosis had slightly higher morbidity rate as compared with Hartmann's stoma, but this difference was not statistically significant. Emergency resection of sigmoid volvulus had a higher rate of postoperative complications (34). The most common morbidity conditions were anastomotic leaks and surgical site infections (4). Retrospective studies showed that a modified RPA and a modified blow-hole colostomy provide satisfactory results and help to avoid these morbid complications (35,36).

Despite a lack of high-quality evidence on the management of gangrenous sigmoid volvulus, this review found that the choice of stoma versus RPA had no different effect on morbidity or mortality, but stoma formation required a relatively less operation time. Low-level evidence and expert opinion suggest that, as long as the patient is stable and tension-free anastomosis is feasible, resection and primary anastomosis can be carried out with acceptable morbidity, mortality, and length of hospital stay.

Although high-level evidence is lacking, numerous small-sample and low-level evidences support resection and primary anastomosis for gangrenous sigmoid volvulus when certain conditions are met, such as intraoperative hemodynamic stability, the absence of gross peritoneal contamination, the ability to achieve a tension-free anastomosis, the presence of viable and well-vascularized bowel ends after resection, and good functional status (22,24-26,31). Gangrenous sigmoid volvulus is classified as group III in the Atamanalp 2020 sigmoid classification, which has the highest risk of mortality (10-60%) and morbidity (5-30%) (32). Atamanalp 2020 recommends resection and anastomosis for patients under the age of 70, ASA I-III, and intact bowel, whereas stoma is recommended for patients over the age of 70, ASA IV-V, and perforated bowel, or borderline ischemia (32). We devised a new protocol considering age, symptom duration, ASA, ECOG, organ dysfunction, cable tension free anastomosis, and the presence of septic shock. We recommended resection and primary anastomosis for patients aged less than 60 years, with ASA I-II, ECOG 0-II, symptom duration less than 72 hours, no organ dysfunction, no septic shock, and WBC count less than 18,000/mm^3^, whereas stoma was recommended for patients aged greater than 60 years, with ASA III-IV, ECOG III-IV, symptom duration more than 72 hours, septic shock with organ dysfunction, and WBC greater than 18,000/mm^3^ (Table1).

**Table 1:** Atalel-Zelalem Proposed a new protocol for management of gangrenous sigmoid volvulus

	Resection and Anastomosis	Stoma	Definitions
Age	<60years	>60years	
ASA class	I-II	III-V	
ECOG	0-II	III-IV	
Duration	<72 hours	>72 hours	
Septic shock	No	Yes	SBP < 90mmHg and which is not responding to 20ml/kg fluid for the first one hour OR requiring vasopressor to maintain SBP above 90mmHg
Organ Dysfunction	No	Yes	Organ Dysfunction defined as Cardiovascular dysfunction: hypotension requiring treatment with dopamine ≥5 ug/kg per min, or any dose of norepinephrine OR Respiratory dysfunction: PaO_2_/FiO_2_ ratio <300 OR Neurological dysfunction: decreased level of consciousness OR Renal dysfunction: oliguria, creatinine >2.0 mg/dl OR Hepatic dysfunction: PT-INR >1.5 OR Hematological dysfunction: platelet count <100,000/mm3
Bowel	Intact	Perforated and gross contamination bowel	
Anastomosis	Tension free and well vascular after 5-10cm resection distal to gangrenous bowel	Impossible	
WBC	<18,000	>18,000	

This review is the largest study with acceptable quality to provide clinically significant important information. First, it disproves the dogma that resection and primary anastomosis is associated with high mortality and morbidity in treatment of gangrenous sigmoid volvulus. Increased mortality and morbidity after surgical interventions have been linked to more heinous factors than just the specific surgical procedure. However, high level evidences are needed for further recommendations.

This review has some limitations. First, this meta-analysis as well as the included articles did not address the contributing factors for mortality and morbidity in each intervention. Second, high quality evidence was lacking in the included articles, which may be due to an ethical conflict in conducting randomized control trials.

In conclusion, this meta-analysis showed that resection and primary anastomosis and Hartmann's stoma had no significant difference in mortality and morbidity for the treatment of gangrenous sigmoid volvulus when performed with selection criteria. Resection and primary anastomosis is advised as the first line treatment for gangrenous sigmoid volvulus under certain conditions such as intraoperative hemodynamic stability, the absence of gross contamination, the capacity to achieve a tension-free anastomosis, the absence of comorbidity, the presence of viable and well-vascularized bowel ends after resection, and good functional status. The findings from this review have a better quality compared with previously available evidences on the management of gangrenous sigmoid volvulus. However, the area requires further extensive research in order to get a high level of evidence especially on examining the contributing factors for morbidity and mortality.
